# Disturbances of mitochondrial dynamics in cultured neurons infected with human herpesvirus type 1 and type 2

**DOI:** 10.1007/s13365-019-00762-x

**Published:** 2019-06-03

**Authors:** Joanna Cymerys, Marcin Chodkowski, Anna Słońska, Małgorzata Krzyżowska, Marcin W. Bańbura

**Affiliations:** 1 Department of Preclinical Sciences, Faculty of Veterinary Medicine, Division of Microbiology, Warsaw University of Life Sciences, Ciszewskiego 8, 02-786 Warsaw, Poland; 2 Military Institute of Hygiene and Epidemiology, Kozielska 4, 01-163 Warsaw, Poland

**Keywords:** HHV-1, HHV-2, Neuronal cell culture, Neurodegeneration, Mitochondrial dysfunction, ROS

## Abstract

Human herpesvirus types 1 and 2 (HHV-1 and HHV-2) are neurotropic viruses which remain latent for life and reactivate to cause recurrent infections. HHV-1 has been found to be involved in accumulation of β-amyloid, hyperphosphorylation of tau proteins, and inflammation in the brain, which can later result in neuronal dysfunction and neurodegeneration. The relationship between HHV-2 and events associated with neurodegeneration has not been extensively studied. Neurons, more than any other cell type, depend on mitochondrial trafficking for their survival, and many types of mitochondrial abnormalities have been described in the etiology of neurodegenerative diseases. Therefore, in this study, we concentrated on mitochondrial dysfunction associated with HHV-1 and HHV-2 infection of primary murine neurons in vitro. We showed that starting from the first stages of HHV-1 and HHV-2 infection, an interaction of viral particles with the mitochondrial network occurs. Both HHV-1 and HHV-2 infection affected mitochondrial function at multiple levels, including upregulation of mitochondrial fission, decrease of the mitochondrial membrane potential, and increase of ROS level. The changes observed in the organization of the mitochondrial network and physiology of productively infected neurons provide appropriate conditions for HHV-1 and HHV-2 replication and are required for effective viral spread.

## Introduction

Herpes simplex virus types 1 and 2 (human herpesvirus types 1 and 2; HHV-1, HHV-2) are ubiquitous, neurotropic pathogens, belonging to the alpha-herpesvirus subfamily. Both viruses can reach the sensory neurons innervating the site of primary infection, and establish a lifelong latent infection. Reactivation from the latent state causes recurrent infections. HHV-1 and HHV-2 are the most common pathogenic cause of sporadic acute encephalitis in humans. Herpesvirus encephalitis is associated with a high mortality rate and significant neurological, neuropsychological, and neurobehavioral sequelae, which afflict patients for life. HHV-1 has been suggested as an environmental risk factor for neurodegenerative diseases (e.g., Alzheimer’s disease (AD)) (Santana et al. 2012, 2013; Wozniak et al. 2011). The reasons for connecting HHV-1 with AD come from data linking HHV-1 directly to the main neuropathological features of AD: amyloid plaques and neurofibrillary tangles (NFT), which comprise mainly of β-amyloid (Aβ) and abnormally phosphorylated tau protein (Santana et al. 2012; Alvarez et al. 2012; Wozniak et al. 2009). A growing number of studies has also pointed to mitochondrial dysfunctions and oxidative stress as key players in the pathogenesis of neurodegenerative diseases (Murata et al. 2000; Valyi-Nagy and Dermody 2005). HHV-1 has been reported to induce depletion of glutathione, the main antioxidant defense, and to increase reactive oxygen species (ROS) levels and lipid peroxidation (Santana et al. 2013). Mitochondrial dysfunction and neurodegeneration are considered to be two faces of the same coin and an early pathological event in brain dysfunction. Mitochondria are extremely dynamic organelles that are constantly changing their shape, size, and location in response to cellular and environmental cues. The balance between mitochondrial fission and fusion allows for rapid adaptation to meet the energetic demand of neurons. Fusion helps mitigate stress by mixing the contents of partially damaged mitochondria. Fission is needed to create new mitochondria, but it also contributes to quality control by enabling the removal of damaged mitochondria and it can facilitate apoptosis when the cellular stress is at high level (Youle and Bliek 2012; Cid-Castro et al. 2018; Chodkowski et al. 2018).

HHV-1 has been shown to directly or indirectly alter mitochondrial function and dynamics (Murata et al. 2000; Santana et al. 2013; Kramer and Enquist 2012). Other researchers suggested existence of a relation between HHV-2 and neurodegenerative diseases (Kristen et al. 2015). Nevertheless, the mechanisms involved in triggering the neurodegenerative process during HHV-2 infection, related to mitochondrial dysfunction, are still not clarified.

HHV-2 is a closely related virus and it would not be surprising to discover that HHV-2 infection has effects similar to HHV-1 infection upon the processes related with neurodegeneration. Neurodegenerative diseases have common pathological features, such as abnormal protein aggregation, mitochondrial dysfunction, and neuronal degeneration specific for oxidative stress. In this paper, we decided to answer several key questions regarding mitochondrial function in cultured murine neurons infected with HHV-1 and HHV-2. The results presented here suggest that abnormal mitochondrial dynamics and dysfunction, including increased levels of ROS and reduced ΔΨ (mitochondrial membrane potential), are probably associated with neuronal dysfunction as the result of productive infection with HHV-1 and HHV-2. The changes in the organization and functioning of mitochondria observed in productively infected neurons contribute to HHV-1 and HHV-2 replication.

## Materials and methods

### Neuron culture

Balb/c (H-2d) mice were used to establish primary culture of murine neurons. Pregnant female mice (16–19 days after mating) were sacrificed; fetuses were removed and decapitated for brain collection. Cerebral hemispheres were isolated from the fetal brains, washed three times in cold HBSS solution (10× Hanks Buffer; Life Technologies), and then incubated in 2.5% trypsin solution (Life Technologies) at 37 °C for 15 min. Brain cells suspension was washed three times in warm HBSS solution and dissociated with a pipette. The cells were then counted and suspended in inoculating fluid (plated onto poly-L-lysine, or poly-D-lysine with laminin-coated coverslips and plates at a density of 5 × 10^3^ to10^4^ neurons per well). Neuronal cells were cultured in B-27 Neuron Plating Medium consisting of neurobasal medium, B27 supplement, glutamine (200 mmol/L), glutamate (10 mmol/L), antibiotics (penicillin and streptomycin, 1%), 5% fetal bovine serum, and 5% equine serum (Life Technologies). To eliminate non-neural cells, cultures were maintained in growth medium supplemented with 10 μM cytosine β-D-arabinofuranoside (after 3 days for 24 h) (Sigma-Aldrich). Next, the medium was removed and replaced with Neuron Feeding Medium (B-27 Neuron Plating Medium without glutamate). In this medium, murine neurons were maintained for the next 6 days prior to treatments, at 37 °C with 5% CO_2_.

**Fig. 1:**
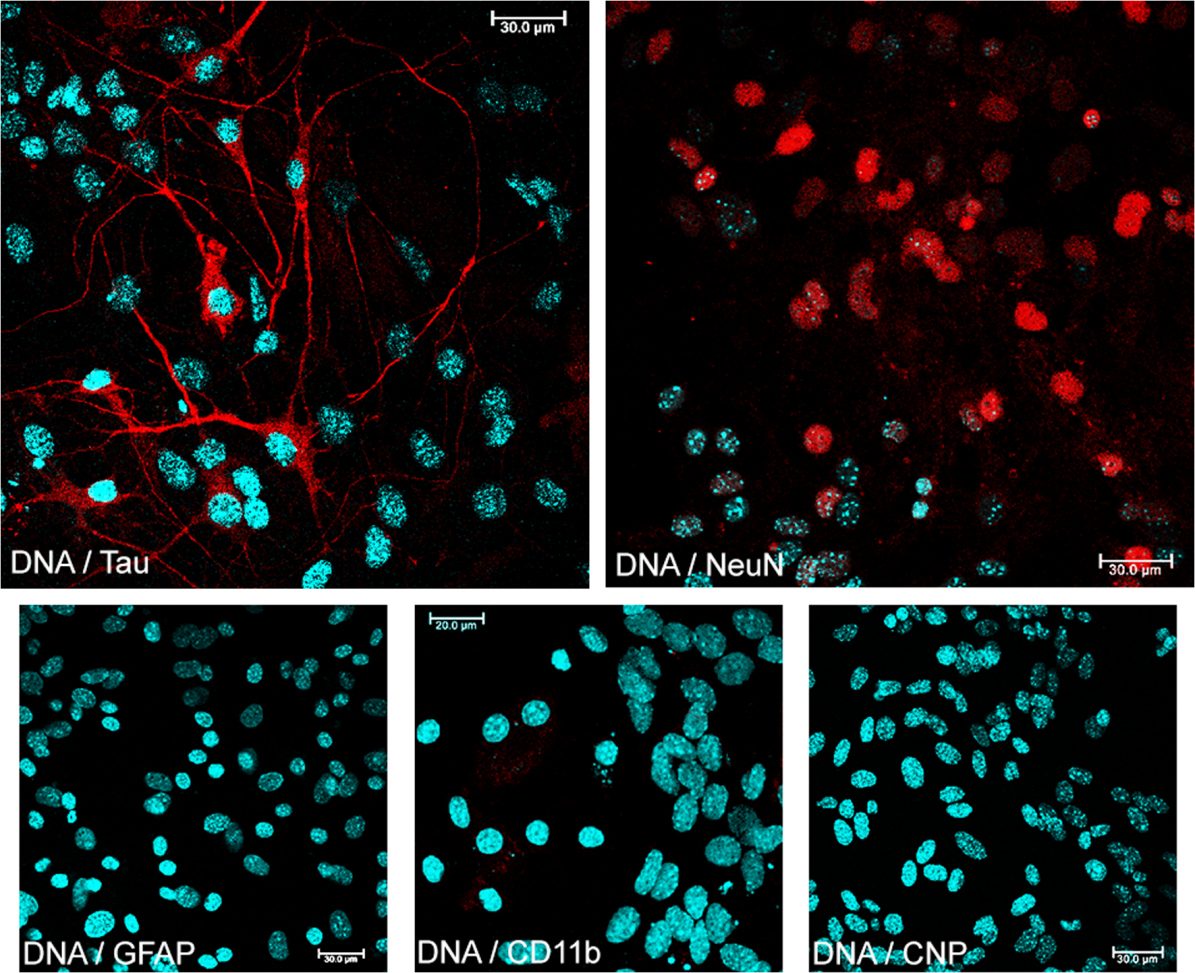
Phenotyping of primary culture of murine neurons. Immunofluorescence labeling with specific mouse mAb: anti-NeuN antibody—neuronal marker (red neuronal nucleus); anti-Tau5 (red neuronal cytoplasm); anti-GFAP (astrocytes, red); anti-CNP (oligodendrocytes, red); anti-CD11b (microglia, red); and nucleus—blue

Treatment with cytosine β-D-arabinofuranoside consistently yields cultures with more than 95% of cells being neurons as judged by cell morphology and labeling with specific mouse mAb: (i) anti-NeuN antibody-Neuronal marker (Alexa Fluor 647), (dilution 1:50; Abcam); anti-Tau5 (for neurons; 1:100; Life Technologies), (ii) anti-GFAP (for astrocytes; dilution 1:100; Calbiochem), (iii) anti-CNP (for oligodendrocytes; dilution 1:100; Merck), and (iv) anti-CD11b (for microglia; dilution 1:100; BD Biosciences), to confirm the cell type (Fig. 1). Tau5, GFAP, and CNP antigens were detected with Texas Red-X goat anti-mouse IgG (1:1000; Life Technologies) and CD11b was detected with Streptavidin-Alexa 647 (1:200; Life Technologies). Cell nuclei were stained with Hoechst 33258 according to the manufacturer’s recommendations (1 μg/mL). Results were evaluated using a Leica white light laser scanning confocal microscope (Leica TCS SP8-WWL, KAWA.SKA Sp. z o.o., Poland) with a × 63 oil-immersion lens. Images were captured and converted to 24-bit tiff files for visualization using the Leica Application Suite X (LAS X) software platform (Leica Microsystems).

### Viruses and inoculation of cells

HHV-1 McIntyre strain and HHV-2 333 strain were grown and titrated in Vero cells (ATCC, no. CRL1587). Vero cells (grown in DMEM; Gibco) were infected with HHV-1 or HHV-2 at a low multiplicity of infection MOI = 0.001 plaque-forming units (PFU)/cell. One hour postinfection (hpi) at 37 °C, the inoculum was aspirated, fresh culture medium was added, and the cells were cultured for 3 days. Culture supernatants were harvested at 72 hpi and after three cycles of freezing (80 °C)/defrosting, centrifuged at 800 g for 10 min, and stored in small volumes at 80 °C.

Primary murine neuronal cells (10^6^ cells per well) were infected with HHV-1 or HHV-2 (MOI = 1) for 60 min at 37 °C. After adsorption, the inoculum was aspirated and fresh culture medium was added. Infected cells were incubated for 2 and 24 h. The TCID_50_ (tissue culture infectious dose) in neuronal cell cultures was 1.06 × 10^5^ for HHV-1 and 1.28 × 10^5^ for HHV-2. The observed differences in cell death-inducing potential between those two types of viruses were minimal and not significant statistically.

### Viral DNA quantification

HHV-1 and HHV-2 DNA in cultured neurons was quantified using real-time PCR (qPCR) with fluorescent probes, complementary for the sequence within the amplified products. At 2 and 24 hpi, viral DNA was isolated from appropriate material (cells or culture medium) using the High Pure Viral Nucleic Acid Kit (Roche Diagnostics, Mannheim, Germany) according to manufacturer’s protocol and analyzed using real-time PCR. To determine the number of virus copies per reaction, standard curve was prepared as described previously (Krzyżowska et al. 2013). Briefly, fragments of glycoprotein B regions from HHV-1 and HHV-2 regions were amplified using appropriate primers for HHV-1: HHV-1Fext (GTGATGTTGAGGTCGATGAAGGT) and HHV-1Rext (ACAACGCGACGCACATCAAGGT) and for HHV-2: HHV-2Fext (CGTACGATGAGTTTGTGTTGGCGA) and HHV-2Rext (TCAGCTGGTGAGAGTACGCGTA). The products were cloned in pGEM-T Easy Vector (Thermo Fisher Scientific). Serial dilution of recombinant plasmids was prepared ranging from 10 to 10^7^ copies per reaction. The real-time PCR were performed in 96-well plates using a 7500 Real Time PCR System thermocycler (Applied Biosystems, Foster City, CA, USA) with TaqMan Universal Master Mix II (Applied Biosystems) using primers and probe labeled with JOE, as described previously (Namvar et al. 2005).

### Immunofluorescent labeling and microscopy analysis

Primary murine neurons seeded on glass coverslips in a six-well plate were infected with HHV-1 or HHV-2. At 2 and 24 hpi, cells were incubated for 30 min at 37 °C with 100 nM MitoRed (Sigma-Aldrich, St. Louis, MO, USA) to visualize the mitochondrial network morphology and distribution. After incubation, cells were washed three times with warm (37 °C) culture medium for 5 min. To detect HHV-1 and HHV-2 antigens or dynamin-related protein 1 (Drp1), cells were fixed with 3.7% PFA in PBS for 20 min. Fixed cells were then permeabilized with 0.5% Triton X-100 (Sigma-Aldrich) in PBS (15 min) and blocked with 1.5% bovine serum albumin (BSA, Sigma-Aldrich) in 0.1% Triton X-100-PBS solution (30 min) to prevent nonspecific binding. The presence of Drp1 was detected by using DNM1L polyclonal antibody (dilution 1:500; Thermo Fisher Scientific) and Alexa Fluor 488 goat anti-rabbit (dilution 1:250; Thermo Fisher Scientific). The presence of viral antigen was detected by using rabbit mAb anti-HSV (dilution 1:250; Dako) and anti-rabbit FITC (dilution 1:200; Thermo Fisher Scientific). Cell nuclei were stained with Bisbenzimidine/Hoechst 33258 (1 μg/mL), in compliance with the manufacturer’s recommendations. Slides were mounted in ProLong Gold Antifade Reagent (Thermo Fisher Scientific). Non-infected neurons served as negative control.

Images were acquired using a Leica white light laser scanning confocal microscope (Leica TCS SP8-WWL, KAWA.SKA Sp. z o.o., Poland) with a × 63 oil-immersion lens. Images were captured and converted to 24-bit tiff files for visualization using the Leica Application Suite X (LAS X) software platform (Leica Microsystems).

### Analysis of mitochondrial morphology

For analysis of mitochondrial morphology, MiNa Single Image macro was used. This tool allows to compute number of individuals, number of networks, mean length of branches/rod, mean network size, mean network size per branches, and mitochondrial footprint. In order to perform this analysis, images obtained from confocal microscopy were used according to the protocol of Valente et al. (2017).

### Image cytometry analysis

The NucleoCounter NC-3000 image cytometer (ChemoMetec, Denmark) was used for mitochondrial membrane potential determination and neurons vitality evaluation after HHV-1/HHV-2 infection. Using Mitochondrial Potential Assay, the cultured neurons were stained with JC-1 (cationic dye 5,5,6,6-tetrachloro-1,1,3,3-tetraethylbenzimidazol-carbocyanine iodide; ChemoMetec A/S). First, suspended cells (non-infected and at 2 and 24 hpi) were diluted with PBS to a final concentration of 1.5 × 10^6^ cells/mL. The samples were then incubated with 12.5 mL of 200 mg/mL JC-1 for 10 min at 37 °C. After the incubation, the samples underwent two washing procedures and at the end of the second washing, samples were resuspended in 250 mL of 1 mg/mL 4′,6-diamidino-2-phenylindole in PBS. Subsequently, the samples were analyzed with the NucleoCounter NC-3000, according to the manufacturer’s protocols. Using Vitality Assay (analysis of the level of cellular thiols), cultured neurons were stained with VitaBright-48 (ChemoMetec A/S), acridine orange (ChemoMetec A/S), and propidium iodide (PI; ChemoMetec A/S). First, the suspended cells (non-infected, 2 and 24 hpi) were diluted with PBS to a final concentration of 2.0 × 10^9^ cells/mL and were mixed with 5 mL of VitaBright-48•PI•acridine orange. Subsequently, the samples were analyzed with the NucleoCounter NC-3000, according to the manufacturer’s instructions. The results were analyzed using the NucleoView NC-3000 software (details of the NucleoCounter NC-3000 design and capabilities are available at www.chemometec.com).

A positive control for mitochondrial potential analysis was prepared by adding CCCP (carbonyl cyanide m-chlorophenyl hydrazone; 5 μl/ml cell culture medium). Non-infected neurons served as negative control.

### Flow cytometry analysis

Flow cytometry analysis was used to measure mitochondrial mass in HHV-1- and HHV-2-infected neurons. All cytometric analyses were performed on live, non-sustained cells. Neurons (10^6^ cells/mL) at 2 and 24 hpi were stained with mouse MitoTracker Green FM (200 nM; Thermo Fisher Scientific) for 10 min in 37 °C, according to the manufacturer’s protocols. MitoTracker Green FM is a non-fluorescent dye in aqueous solutions, but becomes fluorescent once it accumulates in the lipid environment of mitochondria, regardless of membrane potential. Samples were analyzed by BD LSR Fortessa cytometer (BD Biosciences, Franklin Lakes, NJ, USA). Non-infected neurons stained with MitoTracker Green FM served as positive control. Non-infected neurons unstained with MitoTracer Green FM served as negative control.

### Measurement of reactive oxygen species level

Reactive oxygen species (ROS) levels were measured with the CellROX® Green Reagent (Thermo Fisher Scientific), a fluorogenic probe for measuring oxidative stress in live cells (Ex/Em~485/520 nm). Control and HHV-1/HHV-2-infected neurons were stained with 5 μM CellROX® Green Reagent and Hoechst 33342 by adding the probe to the complete media and incubating at 37 °C for 30 min. Next, cells were washed with PBS and then analyzed using a confocal microscope (Fluoview FV10i, Olympus, Tokyo, Japan) with a × 60 water immersion lens. Images were captured and converted to 24-bit tiff files for visualization using the FV10i software (Olympus). Uninfected neurons treated with 1 mM H_2_O_2_ were used as a positive control.

### Determination of ATP levels

ATP levels were measured spectrophotometrically using an ATP Colorimetric/Fluorometric Assay Kit (Sigma-Aldrich). ATP concentration is determined by phosphorylating glycerol, resulting in a colorimetric (570 nm) or fluorometric (λex = 535/λem = 587 nm) product proportional to the amount of ATP present. Non-infected and HHV-1/HHV-2-infected neurons (2 and 24 hpi) were collected and treated according to specifications in the ATP Assay Kit protocol. The absorbance of samples was measured using an Epoch Microplate Spectrophotometer (BioTek, Winooski, VT, USA) at OD 570 nm and the results were computed from the standard curve.

### Western blot analysis

Cultured neuronal cells prepared as described before were first washed with ice-cold PBS and lysed in N-PER Neuronal Protein Extraction Reagent (Thermo Fisher Scientific) containing protease and phosphatase inhibitors (Halt Phosphatase Inhibitor Cocktail and Halt Protease Inhibitor Cocktail; Thermo Fisher Scientific), 20 min on ice. The lysates were clarified by centrifugation for 15 min at 4 °C. Quantitation of the protein content in lysates was performed with the Micro BCA Protein Assay Kit (Thermo Fisher Scientific) and spectrophotometry on an Epoch BioTek spectrophotometer. Samples containing 20 μg of protein were incubated with Lammeli sample buffer containing β-ME (Bio-Rad; Hercules, CA, USA) for 5 min at 95 °C. Subsequently, the samples and protein markers were electrophoresed on a 10% polyacrylamide Bis-Tris Plus gel with MES running buffer and transferred onto a PVDF membrane. The membrane was blocked with 5% BSA in TBST and incubated overnight with DNM1L polyclonal antibody (Thermo Fisher Scientific). After several washes in 0.1% Tris-buffered saline (TBS)-Tween 20, blots were incubated with HRP-conjugated secondary antibodies for 1 h at RT and developed using enhanced chemiluminescence (Clarity Western ECL Substrate; Bio-Rad). The protein bands were visualized by ChemiDoc™ MP Imaging System (Bio-Rad, Hercules, CA, USA). Glyceraldehyde 3-phosphate dehydrogenase (GAPDH) was used as a loading control and for protein normalization during densitometry measurements.

### Statistical evaluation

The results were statistically evaluated by one-way analysis of variation (ANOVA) using Student–Newman–Keuls multiple comparisons test and Turkey–Kramer multiple comparisons test. Data were analyzed using the GraphPad Prism™ version 4.03 software (GraphPad Software Inc., San Diego, CA, USA). Statistical differences were interpreted as significant at *p* ≤ 0.05* and highly significant at *p* ≤ 0.01**.

## Results

### The HHV-1 and HHV-2 replication cycle in primary murine neurons

**Fig. 2:**
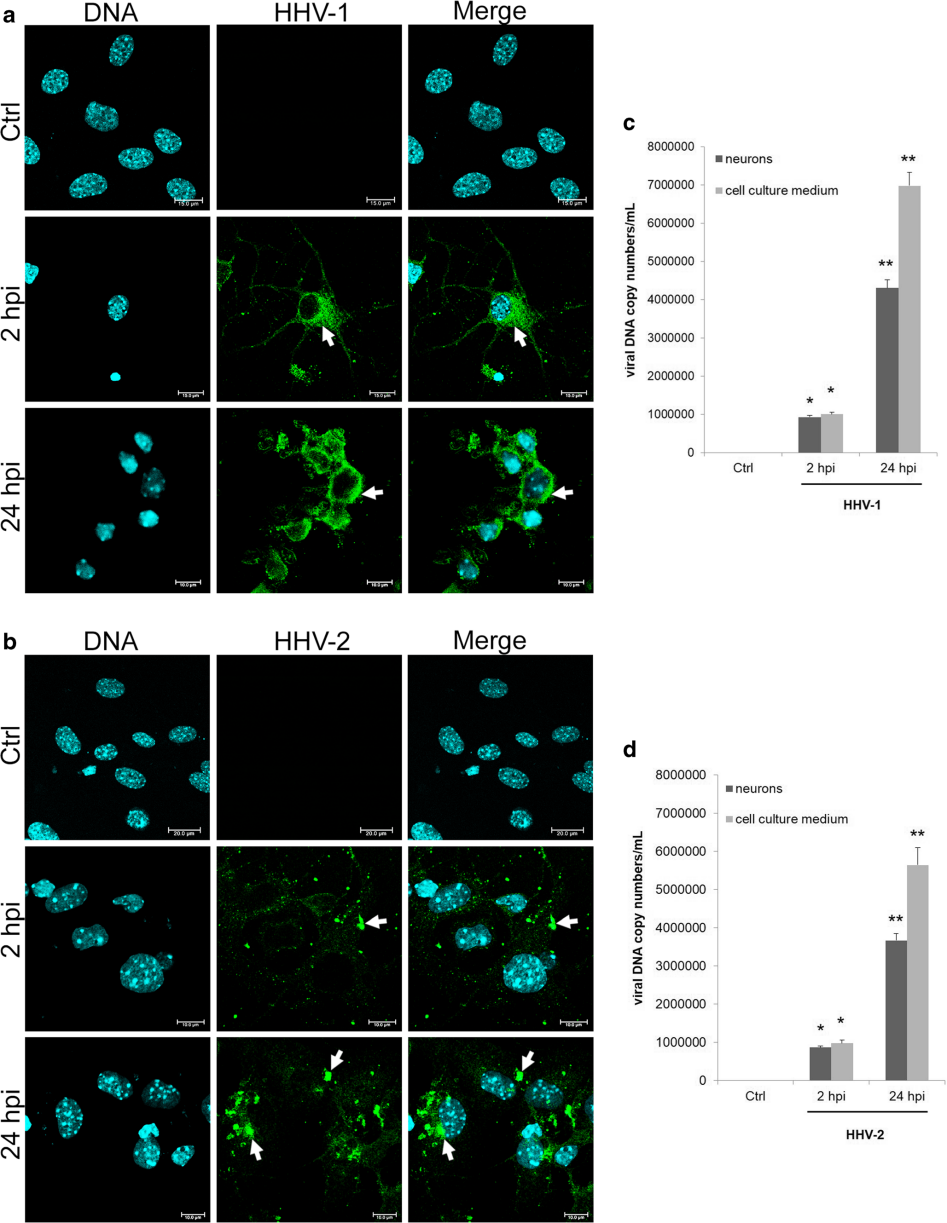
HHV-1 and HHV-2 replication in cultured neurons. Fluorescence images of mock- or HHV-1- (**a**) and HHV-2- (**b**)-infected neurons (2 and 24 hpi). Arrows indicate the accumulation of viral antigens (green fluorescence). (**c**) Real-time PCR analysis of viral DNA copy number in neurons and cell medium during HHV-1 and HHV-2 infection. Statistical differences were interpreted as significant at *p* ≤ 0.05 (*) and *p* ≤ 0.01 (**)

Our previous study has shown that HHV-1 is able to productively infect primary murine neurons with subsequent release of progeny virions (Cymerys et al. 2013). In the present study, we compared the effects of HHV-1 and HHV-2 replication in cultured neurons. Antigens of HHV-1 and HHV-2 were observed already at 2 hpi (early stage of infection) (Fig. 2a, b). The signal was localized mostly in the cytoplasmic compartment. Then, after the full replication cycle, we detected accumulation of viral antigen around the nucleus (especially during HHV-1 infection) and antigens accumulation in cell body and dendrites during HHV-2 infection (Fig. 2a, b; arrows). At 24 hpi, we observed that infection of cultured neurons with HHV-1 and HHV-2 induces the CPE (cytopathic effect) which was manifested by changes in cell morphology and nuclei degeneration (Fig. 2a, b).

The quantitative PCR analysis showed a significant increase of the DNA copy number of analyzed viruses, in comparison to the uninfected control (Fig. 2c, d). The highest, significant increase in the copy number of viral DNA in neurons was observed at 24 hpi with HHV-1 and HHV-2 (4.3 ± 1.01 × 10^6^ copies/ml and 3.6 ± 1.31 × 10^6^ copies/ml respectively; *p* ≤ 0.01). We also found a significant increase in the viral DNA copy number in the culture medium at 24 hpi (6.9 ± 2.07 × 10^6^ copies/ml for HHV-1 and 5.6 ± 1.1 × 10^6^ copies/ml for HHV-2; *p* ≤ 0.05), which was most probably the result of progeny virions release from cells (Fig. 2c, d).

**Fig. 3:**
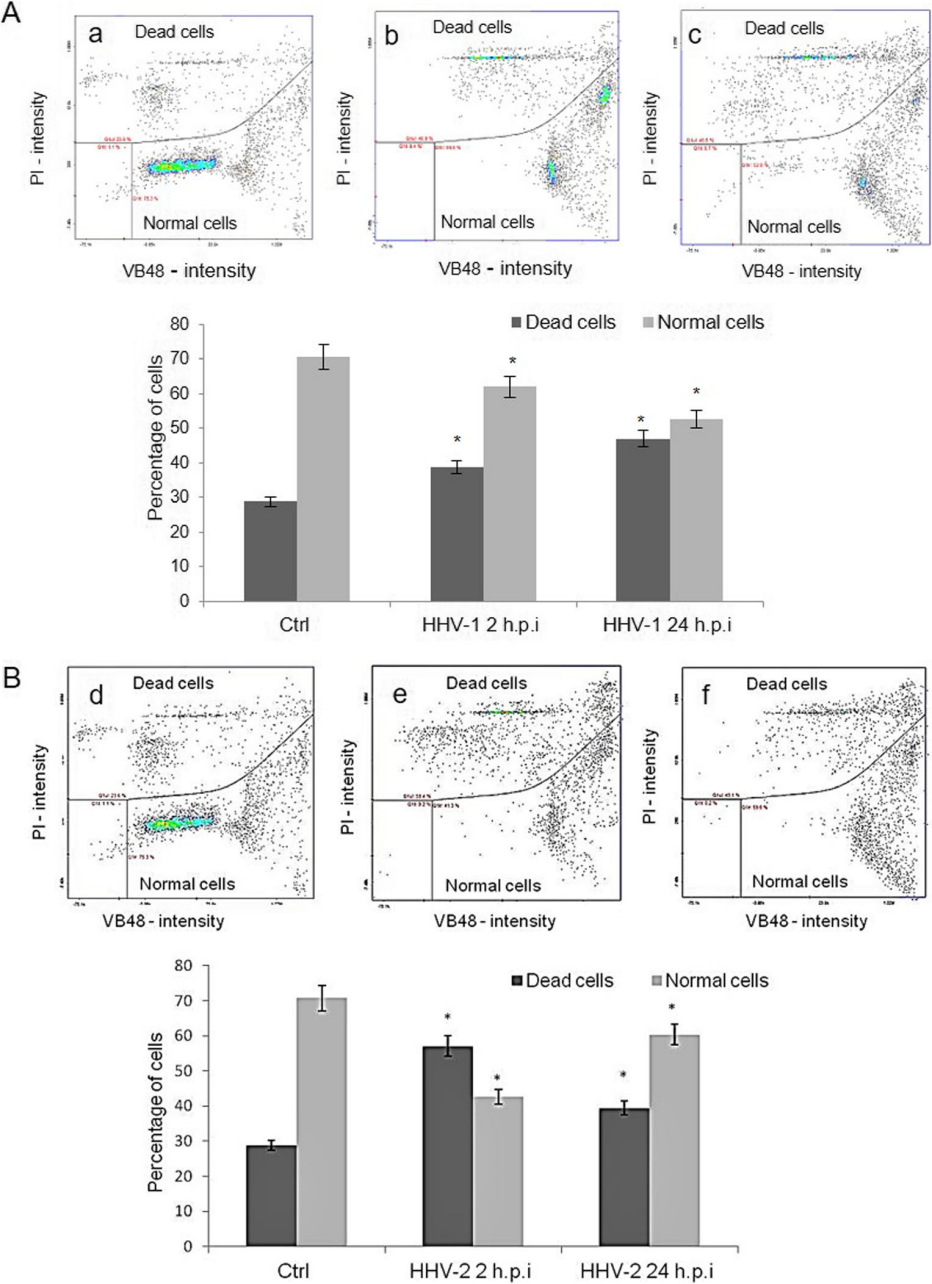
Neuron viability in control cells (**a**, **d**) and after HHV-1 (**A**; **b**—2 hpi, **c**—24 hpi) and HHV-2 (**B**; **e**—2 hpi, **f**—24 hpi**)** infection. Cultured neurons were stained with VitaBright-48™ (VB-48™), acridine orange (AO), and propidium iodide (PI). Representative fluorescence dot plots of image cytometry analysis (**A**, **B**; **a**–**f**) and data from three independent experiments, analyzed by using the NucleoView NC-3000 software, are presented in graphs. Statistical differences were interpreted as significant at *p* ≤ 0.05 (*)

In support of the above observations, we decided to evaluate the neurons’ viability after HHV-1 and HHV-2 infection, using the NucleoCounter NC-3000. Using performing Vitality Assay kit (analysis of the intracellular levels of free thiols, accompanying apoptosis or cell damage), cultured neurons were stained with VitaBright-48, acridine orange, and propidium iodide. It has been found that both HHV-1 and HHV-2 reduced viability of cultured murine neurons (Fig. 3a, b). Viability of uninfected neurons was maintained at 70–75%. After HHV-1 infection, a decrease of 62% in the early stage of infection was observed, followed by additional decrease to approximately 52% within 24 hpi. After infection with HHV-2, a similar effect was observed, the viability dropped to 42% at 2 hpi and to 58% at 24 hpi (Fig. 3a, b).

### Changes in the mitochondrial network morphology and distribution in neurons during HHV-1 and HHV-2 infection

**Fig. 4:**
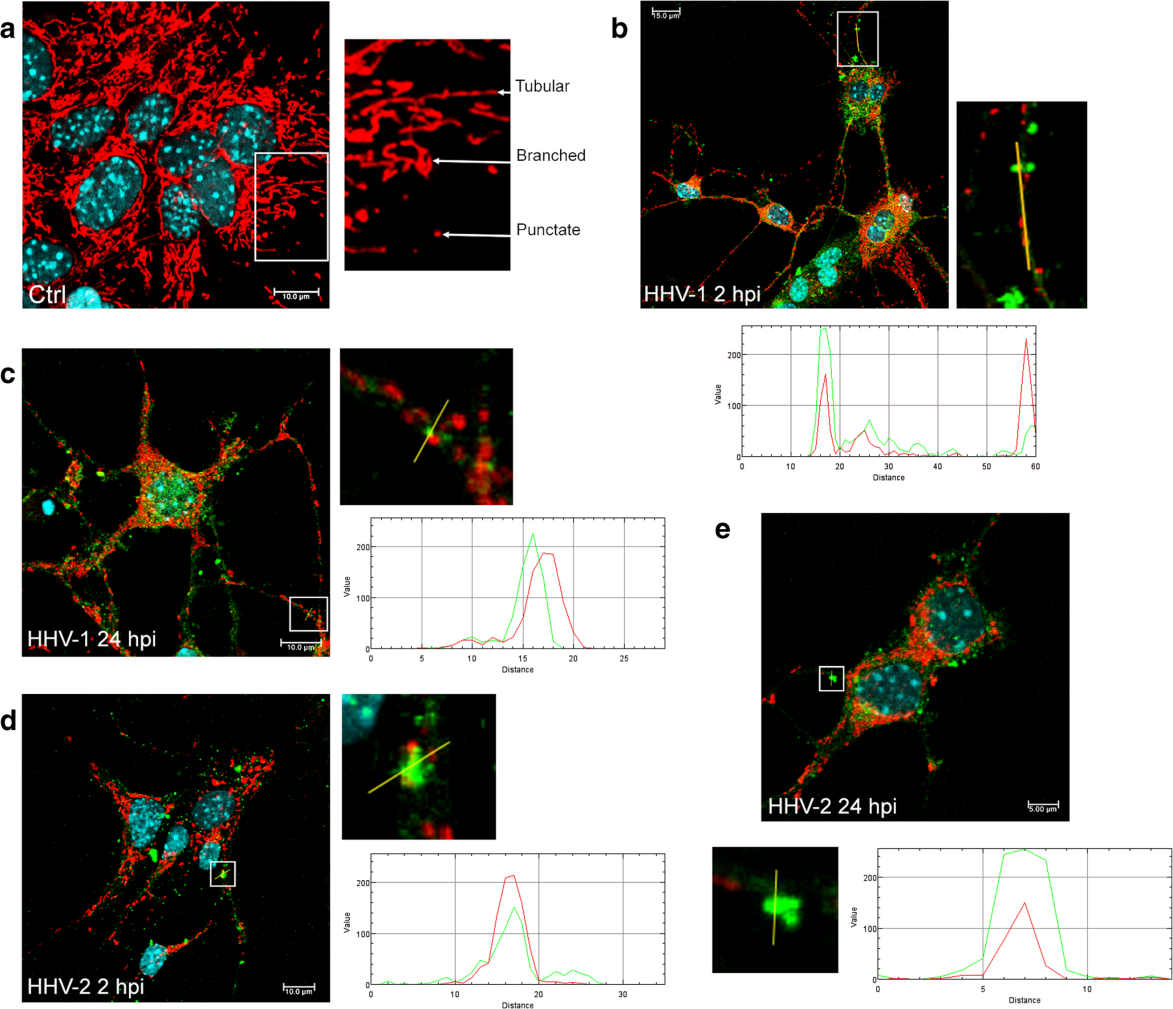
Mitochondrial network morphology in neurons during HHV-1 (**b**, **c**) and HHV-2 (**d**, **e**) infection (2 and 24 hpi). Mitochondria were fragmented and disorganized with the loss of connection between themselves when the branched network was relaxed. Yellow lines indicate fragmented, punctate mitochondria co-localized with a viral antigens (**b**–**e**). The fluorescence intensity of mitochondria (red line) and viral antigens (green line) was measured along the yellow lines (**a**). Uninfected neurons with many tubular, long, and highly interconnected mitochondria. White arrows indicate three types of mitochondrial shape: tubular, punctate, and branched mitochondrial network. Mitochondria—red fluorescence, viral antigens—green fluorescence, nuclei—blue fluorescence

In the uninfected neurons, mitochondrial network was dense, branched, and spread evenly throughout the cell (Fig. 4a). We distinguished three types of mitochondrial shape: tubular, punctate, and branched mitochondrial network. We observed many tubular, long, and highly interconnected mitochondria localized in the subcellular region and a small number of punctate mitochondria (Fig. 4a).

HHV-1 and HHV-2 infection caused changes in the mitochondrial network morphology. Starting from 2 h of HHV-1 or HHV-2 infection, we observed an interaction of viral antigens with the mitochondrial network. The viral antigens were located near the cell nucleus and co-localized with mitochondria. Moreover, accumulation and co-localization of viral antigens with mitochondria inside the long neuronal fibers was observed (Fig. 4b–e). At 24 h of HHV-1 infection (Fig. 4c), we observed changes in the shape of mitochondrial network and distribution within the cell. Mitochondria were fragmented and disorganized with the loss of connection between themselves, when the branched network was relaxed (Fig. 4c). The similar results were obtained for the infection with HHV-2. Starting from 2 hpi, the majority of mitochondria were tightly clustered in close association with viral antigens of multinucleate cells (Fig. 4d). Some mitochondria were irregularly dispersed within the cytoplasm and displayed various forms. The short, non-connected mitochondrial tubules and punctate mitochondria were observed. The manifestation of appearance of progeny virions within the cytoplasm and the co-localization with the mitochondrial structures were observed at 24 hpi (Fig. 4e).

**Fig. 5:**
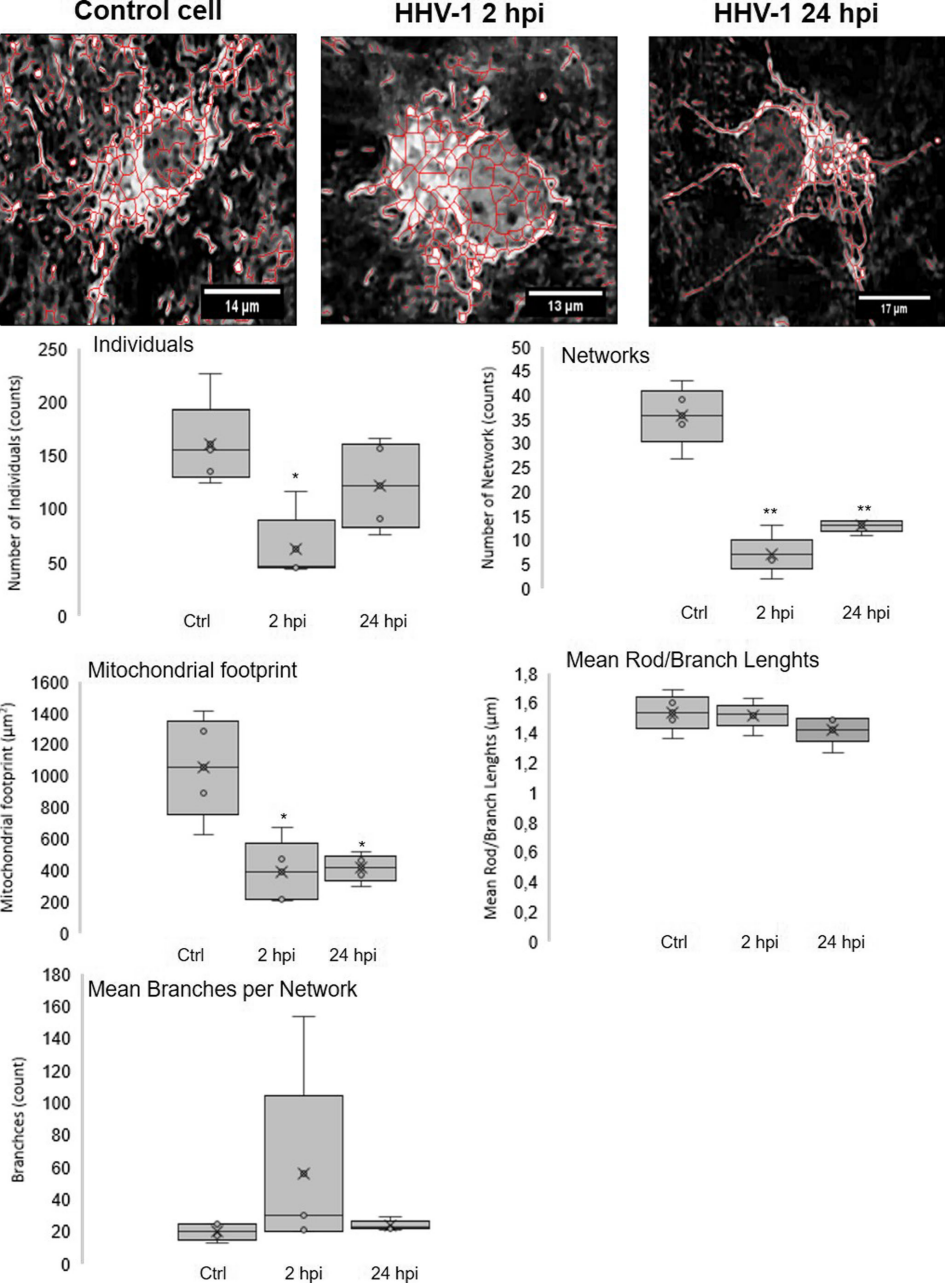
Characterization of mitochondrial morphology of neurons during HHV-1 infection with using MiNa Single Image macro. The number of individuals, number of networks, mean length of branches/rod, mean network size, mean network size per branches, and mitochondrial footprint were examined. The graph shows a summary statistic of infected neurons and control cells (each analysis was performed on 10 cells). Box plot show median (horizontal lines), first to third quartile (box), and the most extreme values (%). Statistical differences were interpreted as significant at *p* ≤ 0.05 (*) and *p* ≤ 0.01 (**)

**Fig. 6:**
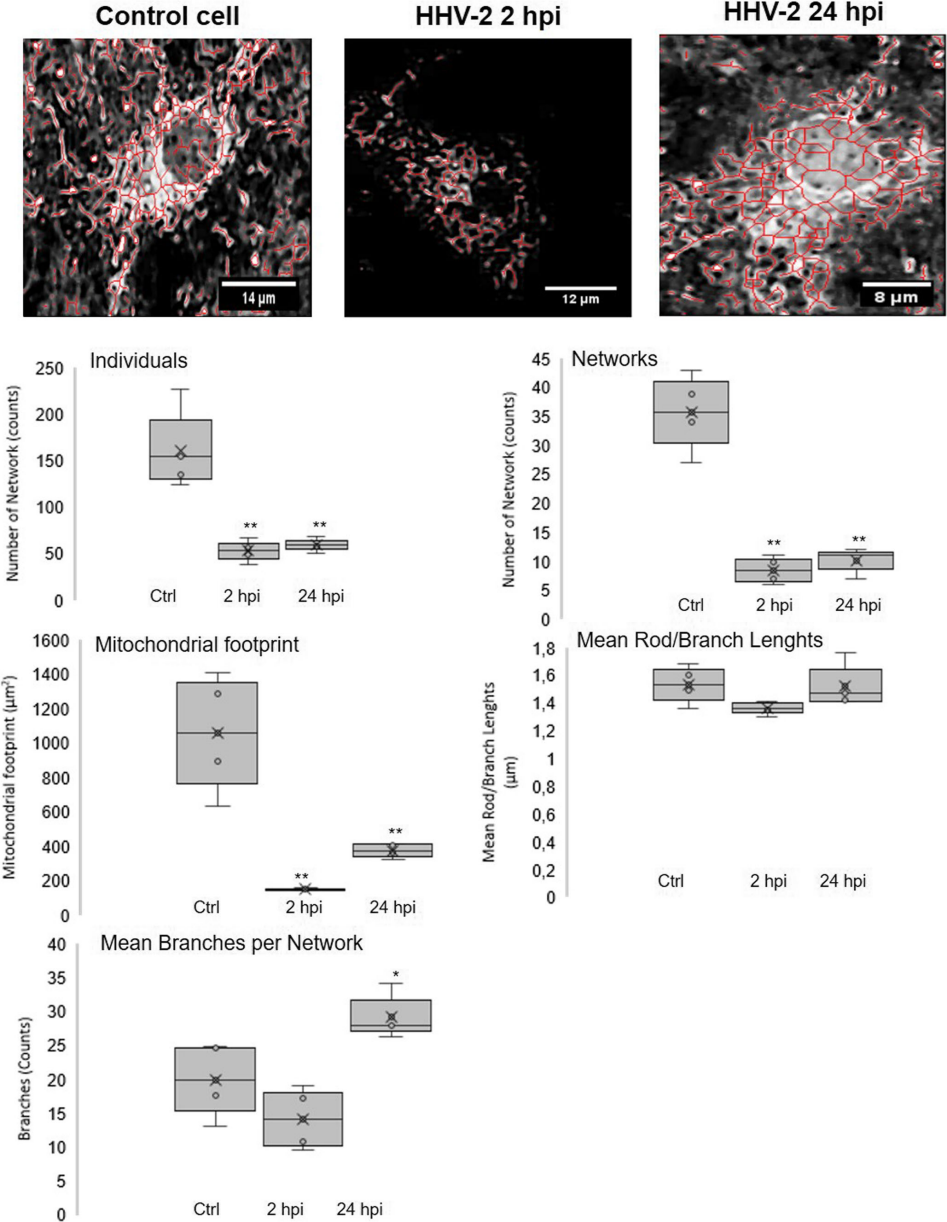
Characterization of mitochondrial morphology of neurons during HHV-2 infection with using MiNa Single Image macro. The number of individuals, number of networks, mean length of branches/rod, mean network size, mean network size per branches, and mitochondrial footprint were examined. The graph shows a summary statistic of infected neurons and control cells (each analysis was performed on 10 cells). Box plot show median (horizontal lines), first to third quartile (box), and the most extreme values (%). Statistical differences were interpreted as significant at *p* ≤ 0.05 (*) and *p* ≤ 0.01 (**)

As indicated in Figs. 5 and 6, we performed an additional characterization of mitochondrial morphology of single neuronal cell on the basis of confocal microscope images. At 2 h of HHV-1 infection, we observed a decrease in a number of mitochondrial networks, and a decrease in the number of individual objects. In addition, the percentage of cross-linked mitochondria and the length of the network branches decreased. Moreover, the total area of mitochondria was significantly reduced (*p* ≤ 0.05) (Fig. 5).

At 24 hpi, the number of objects and number of mitochondrial network increased, in comparison to 2 hpi, but it was still at a low level in comparison to non-infected neurons. Moreover, we observed the reduction of total mitochondrial area. The mean number of branches per network was at the same level as measured in control cells (Fig. 5).

HHV-2 infection caused similar changes as infection with HHV-1 (Fig. 6). We detected a significant decrease in the number of mitochondria (*p* ≤ 0.01), changes in mitochondrial cross-linking, as well as changes in the length of the mitochondrial branches. The mean number of branches per network decreased at 2 hpi, but significant increased at 24 hpi (*p* ≤ 0.05). Both at 2 and at 24 hpi, a significant decrease in the overall mitochondrial surface was observed (*p* ≤ 0.01) (Fig. 6).

**Fig. 7:**
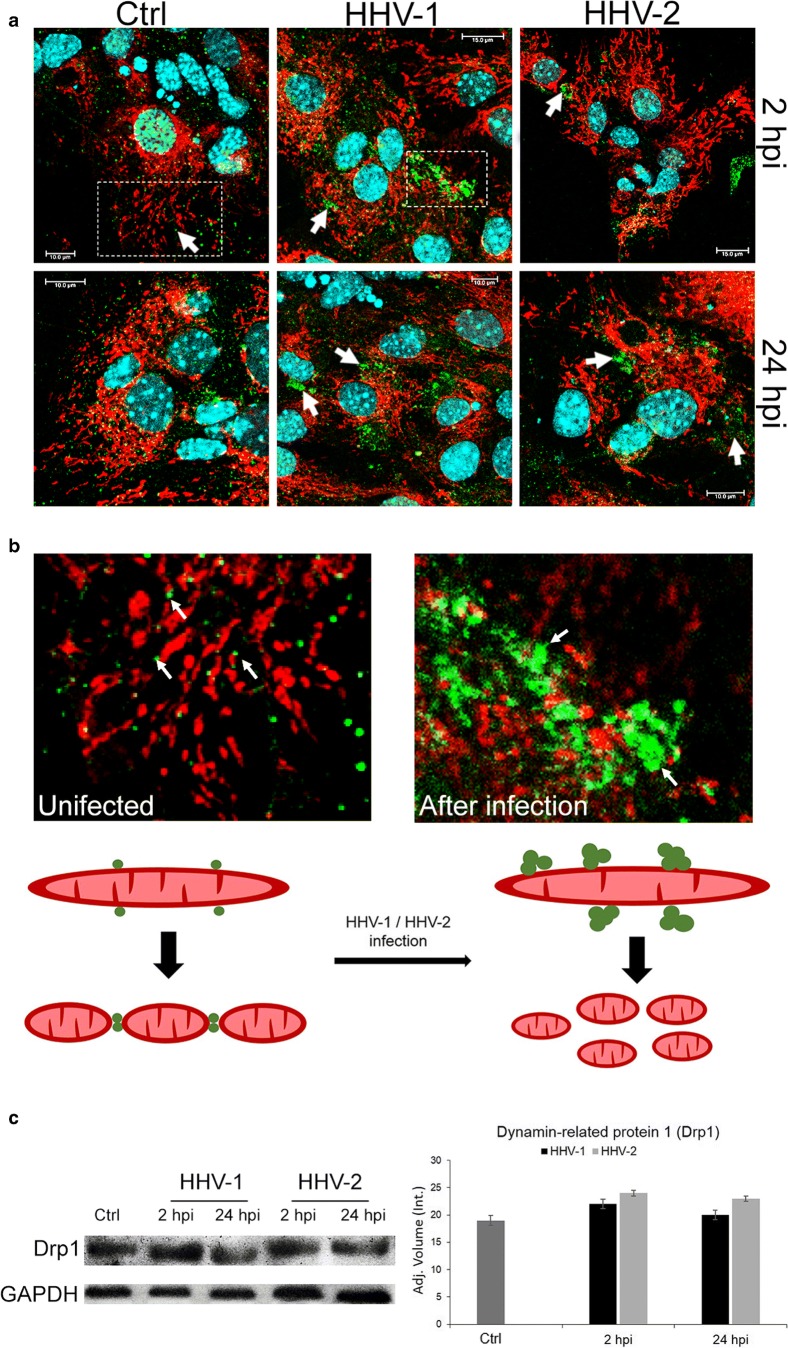
Distribution of Drp1 protein in neurons during HHV-1 and HHV-2 infection. Immunofluorescence labeling was used to examine mitochondrial translocation of the Drp1 fission protein (white arrows indicate localization of Drp1). In uninfected cells, Drp1, as well as the mitochondrial network, remained unchanged and distributed regularly within the cytoplasm (**a**, **b**). During infection with HHV-1 and HHV-2, a progressive fission of the mitochondrial network, as a consequence of accumulation of the Drp1 protein (green fluorescence) on the outer mitochondrial membrane (mitochondria—red fluorescence) was observed (**b**). Representative Western blot analysis and densitometry analysis (from three independent experiments) of the Drp1 protein in HHV-1/HHV-2 infected and uninfected neurons. The level of protein was normalized to GAPDH; results were statistically compared to mock infected control (**c**)

Dynamin-related protein 1 (Drp1) is the most important protein participating in the fission of the mitochondrial network in the cell. Therefore, we decided to investigate the level and distribution of Drp1 in control and HHV-infected neurons. Western blot analysis showed that the level of mitochondrial fission protein remained unchanged in neurons during HHV-1 and HHV-2 infection compared to control, but its distribution within the cytoplasm was altered (Fig. 7c). We found that in uninfected cells, Drp1, as well as the mitochondrial network, remained unchanged and distributed regularly within the cytoplasm. During HHV-1 and HHV-2 infection, Drp1 was partially translocated from the cytoplasm to the outer membrane of the mitochondria (Fig. 7a). This protein has the ability to oligomerize, creating on the surface of mitochondrial membranes spiral structures that, as a result of GTP hydrolysis, tighten and disrupt the integrity of the membranes leading to the mitochondrial fragmentation. We observed this process already at 2 hpi, both for HHV-1 and HHV-2 infection (Fig. 7a, b). During the late stage of infection, with HHV-1 and HHV-2, we found a progressive disintegration of the mitochondrial network, as a consequence of accumulation of Drp1 protein on the outer mitochondrial membrane (Fig. 7b).

### Impact of HHV-1 and HHV-2 infection on mitochondrial physiology

**Fig. 8:**
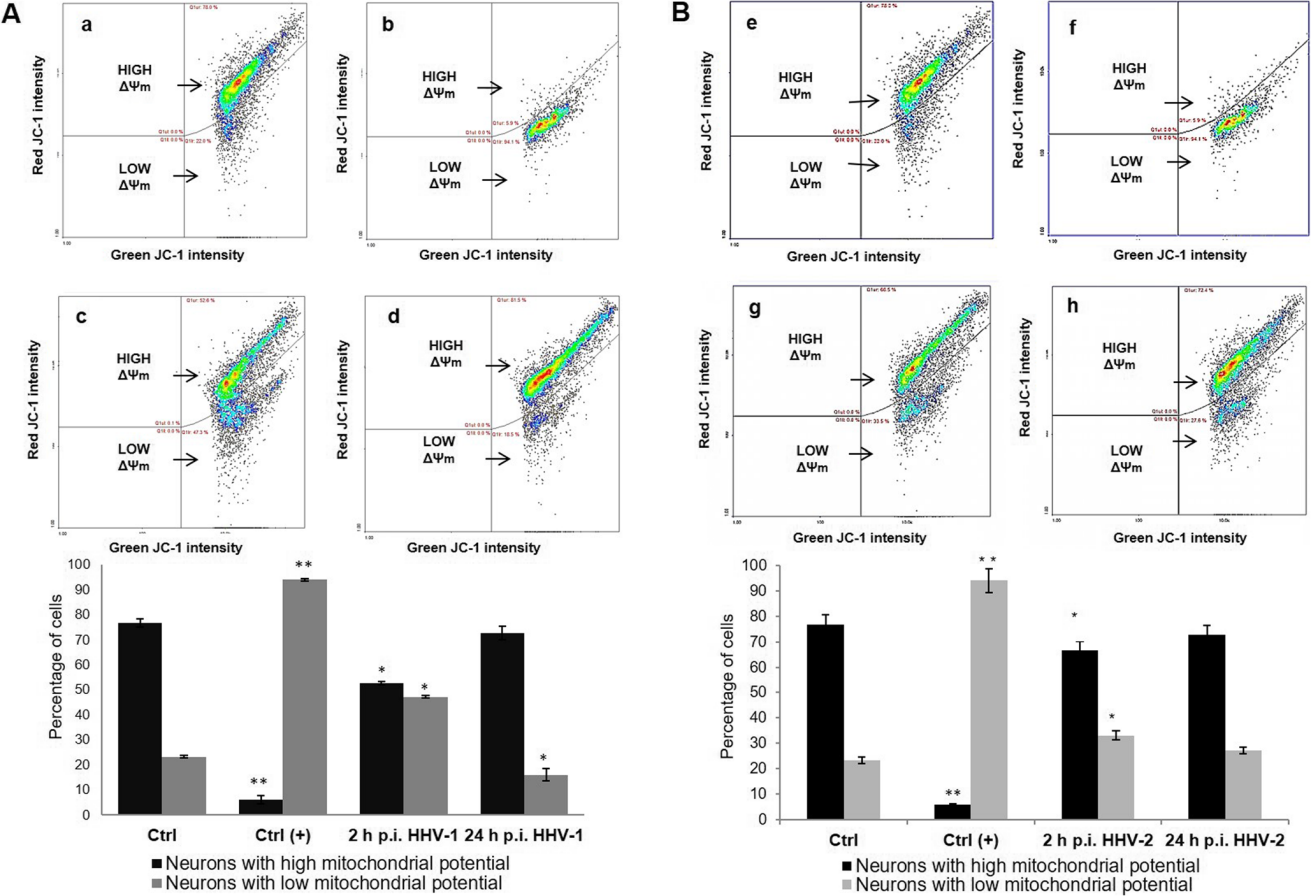
Mitochondrial membrane potential (ΔΨ) in neurons. Representative double fluorescence dot plots of image cytometry analysis (**A**, **B**; **a**–**h**) and data from three independent experiments, analyzed by using the NucleoView NC-3000 software, presented in graphs. Uninfected control cells have a high mitochondrial potential (**A a**, **B e**). CCCP-treated neurons (positive control) have a low mitochondrial potential (**A b**, **B f**). Neurons infected with HHV-1 at 2 hpi (**A**, **c**) and 24 hpi (**A**, **d**); neurons infected with HHV-2 at 2 hpi (**B**, **g**) and 24 hpi (**B**, **h**).The level of green fluorescence in cells is indicated as a percentage. Statistical differences were interpreted as significant at *p* ≤ 0.05 (*) and *p* ≤ 0.01 (**)

For the reason that mitochondrial networks in infected neurons were disorganized and partially fragmented, we hypothesized that infection with HHV-1 and HHV-2 can negatively impact the mitochondrial physiology. Due to the important role of mitochondrial membrane potential (ΔΨ) in mitochondrial homeostasis, we evaluated ΔΨ in uninfected and infected neurons by JC-1 labeling using a NucleoCounter NC-3000 image cytometer (Fig. 8a, b). The principle of the test lies in the fact that JC-1, a mitochondrial potential-sensitive dye, accumulates in the matrix of mitochondria by forming J-aggregates with red fluorescence when the mitochondrial potential is high, and becomes monomer with green fluorescence when it is low. Cultured neurons treated with CCCP, serving as a positive control, demonstrated a low mitochondrial potential (Fig. 8b, f) with dysfunctional mitochondria—JC-1 was present in the cytosol in its monomeric form, emitting green fluorescence. During infection with HHV-1 and HHV-2 (2 and 24 hpi), we observed changes in the mitochondrial potential. The most significant decrease was observed in neurons at 2 hpi (*p* ≤ 0.05). At 24 hpi the decrease of the mitochondrial potential also occurred but it was statistically insignificant and comparable with the values found in the negative control (Fig. 8a, b).

**Fig. 9:**
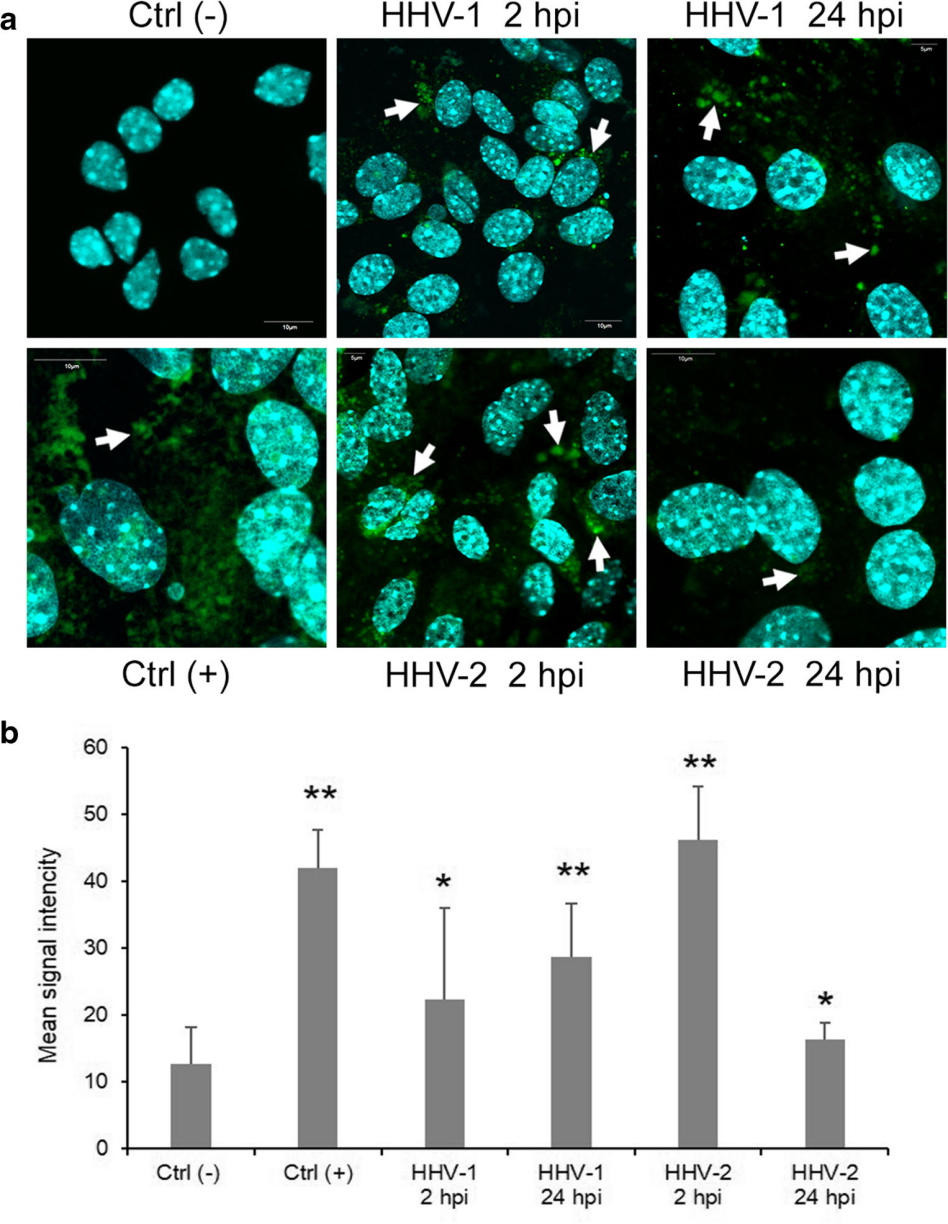
Level of reactive oxygen species (ROS) in neurons during HHV-1 and HHV-2 infection (2 and 24 hpi), measured with the CellROX® Green Reagent. Uninfected neurons treated with 1 mM H_2_O_2_ were used as a positive control (**a**). Data from three independent experiments are presented in graph (**b**). Statistical differences were interpreted as significant at *p* ≤ 0.05 (*) and *p* ≤ 0.01 (**)

Loss of mitochondrial dynamics and reduction in ΔΨ is accompanied by ROS generation. Therefore, in the next step, we measured the cellular ROS level in neurons after HHV-1 and HHV-2 infection. Starting from 2 hpi, there were statistically significant changes in ROS production between infected and control neurons, both for HHV-1 (*p* ≤ 0.05) and HHV-2 (*p* ≤ 0.01) (Fig. 9a, b). The production of ROS was the highest during HHV-2 infection, even higher than in positive control. In addition, during the later stage of HHV-1 and HHV-2 infection (24 hpi), an increased level of ROS was also present, compared to the uninfected neurons (Fig. 9a, b).

**Fig. 10:**
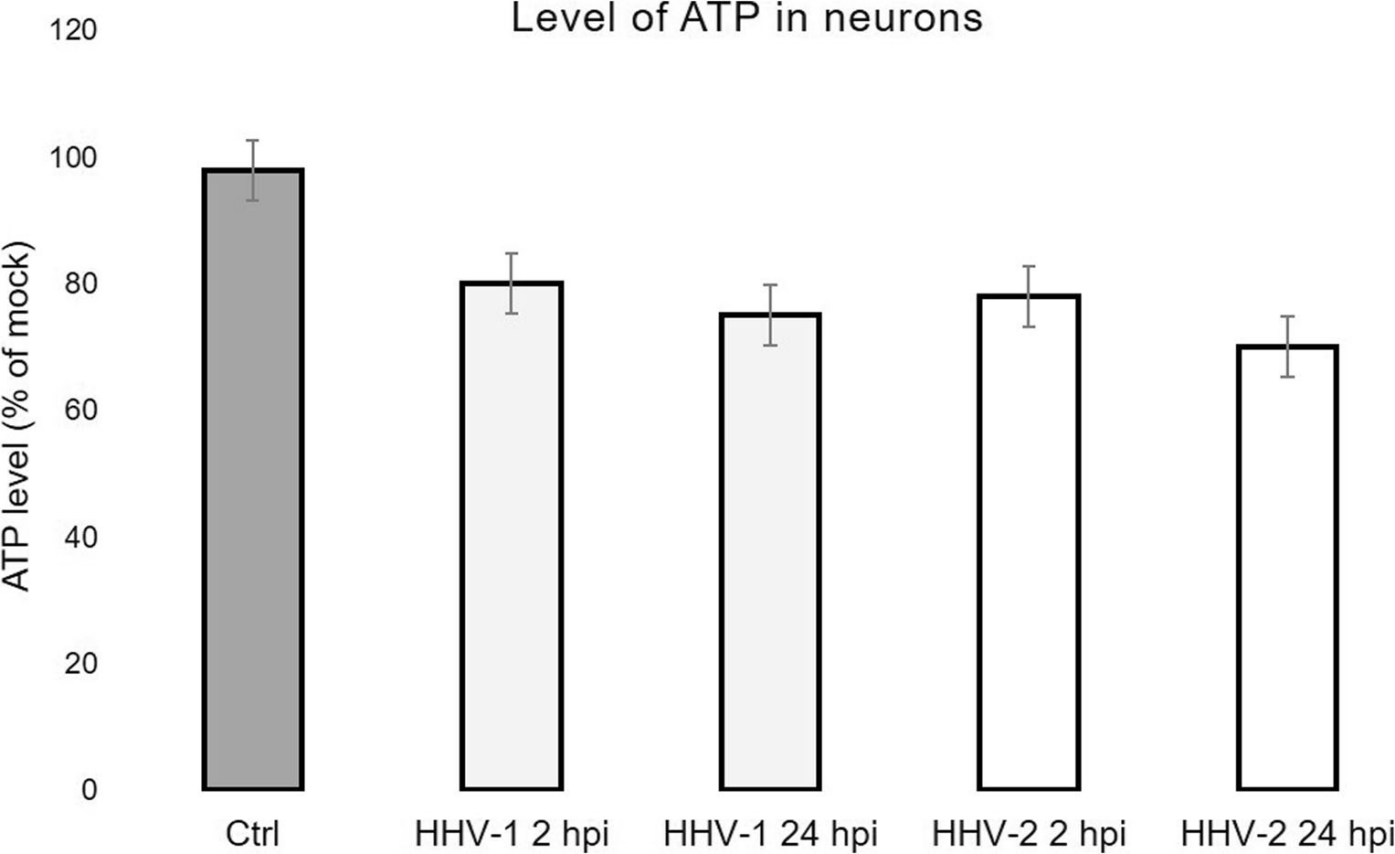
Level of ATP in neurons during HHV-1 and HHV-2 infection (2 and 24 hpi), measured spectrophotometrically using an ATP Colorimetric/Fluorometric Assay Kit

Dysfunction of mitochondrial energy metabolism, resulting from the ΔΨ reduction, leads to reduced ATP production, so we examined how HHV-1 and HHV-2 infection influenced ATP production in neurons. Starting from 2 hpi, the ATP level decreased, in comparison to control. At the late stage of HHV-infection (24 hpi), additional decrease of ATP was recorded, but it was insignificant in comparison to 2 hpi. Generally, the reduction of ATP level in HHV-infected neurons was observed but was statistically insignificant, when compared to non-infected cells (Fig. 10).

**Fig. 11:**
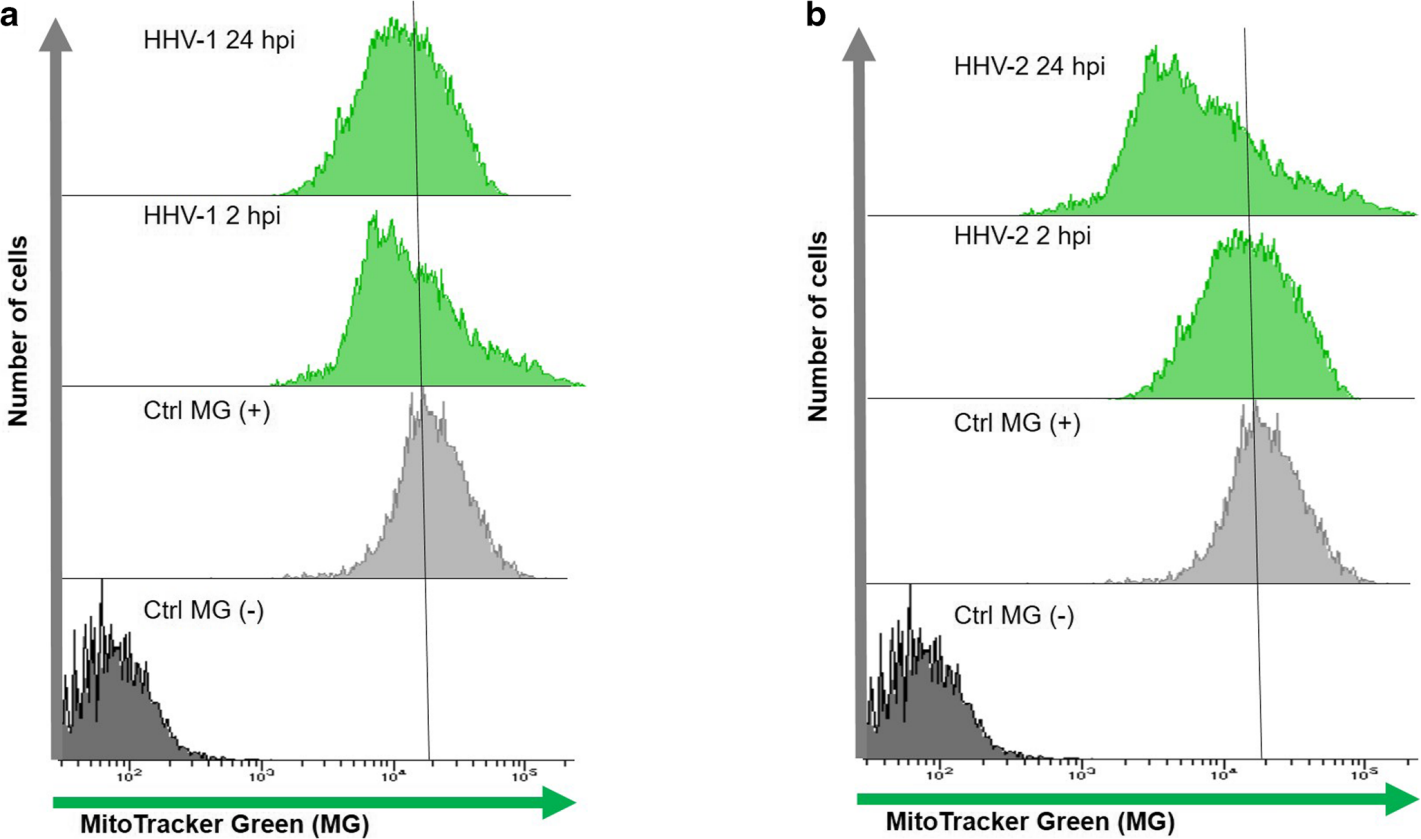
Mitochondrial mass in neurons during HHV-1 (**a**) and HHV-2 (**b**) infection (and 24 hpi). Representative histograms of flow cytometric analysis of MitoTracker Green FM labeling. Non-infected neurons stained with MitoTracker Green FM served as positive control. Non-infected neurons unstained with MitoTracker Green FM served as negative control

Since HHV-infected neurons exhibited a process of mitochondrial fission, next, we decided to determine the mitochondrial mass in such cells. The mitochondrial mass measurement was performed using flow cytometric analysis of live cells with MitoTracker Green FM. At 2 and 24 hpi, there were no statistically significant changes in the mitochondrial mass in neurons infected both with HHV-1 and HHV-2, as compared to positive control (Fig. 11a, b). Only in the case of HHV-2-infected neurons, a slight decrease of mitochondrial mass was observed at 24 hpi (Fig. 11b).

## Discussion

Human herpesvirus types 1 and 2 infections are widespread in human populations all over the world. HHV infections are usually mild but may spread to the central nervous system causing serious neurological disorders. Mounting evidence suggests that HHV-1 can induce neurodegeneration both by direct effects on brain cells and indirect inflammatory and oxidative effects. HHV-1 infection of neuronal cells in vitro has been shown to contribute to development of Alzheimer’s disease (AD). The AD-relevant cellular changes, like β-amyloid (Aβ) accumulation, tau hyperphosphorylation, neuronal, injury and apoptosis, were detected in HHV-1-infected cultured human neuronal cells (Harris and Harris 2015; Wozniak et al. 2009; Alvarez et al. 2012). Some research also point to mitochondrial dysfunction and oxidative stress as playing an important role in the pathogenesis of neurodegenerative diseases (Murata et al. 2000; Valyi-Nagy and Dermody 2005).

HHV-1 and HHV-2 infections in humans are difficult to study; therefore, animal models have been used for better understanding of multiple aspects of HHV pathogenesis. The primary murine neurons culture system proposed by us contributed a great deal to our knowledge of HHV-1 replication and infection (Cymerys et al. 2013). In the present study, we compared the effects of HHV-1 and HHV-2 replication in cultured neurons. The quantitative PCR analysis showed a significant increase in the DNA copy number of analyzed viruses after a full HHV-replication cycle. Furthermore, we demonstrated that during HHV-1 and HHV-2 infection, viability of cultured neurons was reduced by about 20%, in comparison to non-infected cells. In the context of CNS infection with HHV, cell death can function as a cellular antiviral mechanism by depriving the virus of host cells where it replicates, but it can also be a consequence of infection that drives pathology and inflammation.

In general, we observed changes in neuronal morphology characteristic for a productive infection. Our results were obtained in an acute model of infection which is very different from a “milder” latent infection of the brain that periodically follows viral reactivation. However, it is possible to speculate that HHV-1-induced neurotoxic effects, when repeated several times during the lifespan, may lead to the accumulation of “toxic bricks” in neurons, contributing to neurodegeneration independently from other risk factors. People who have more frequent or more extensive peripheral infections can experience an increased reactivation of HHV-1 in the brain and therefore may be at a greater risk of developing neurodegeneration processes or even AD (Martin et al. 2014).

In this study, we examined the interaction between HHV-1 and HHV-2 infection and neurons in the context of the appearance of neurodegeneration markers. Due to the fact that neurons, perhaps more than any other cell type, depend on mitochondrial trafficking for their survival, and many types of mitochondrial abnormalities have been described in the etiology of neurodegenerative diseases, we concentrated on mitochondrial dysfunction.

Mitochondria are the most suitable target for the attack from viruses and the source of ROS produced upon different viral infections. Murata et al. (2000) demonstrated that during HHV-2 infection of epithelial cells, mitochondria cluster around a perinuclear region of the cytoplasm that is enriched with the viral tegument proteins pUL41 and pUL46. Our present study also indicates that starting from the first stages of HHV-1 and HHV-2 infection, the interaction of viral proteins with the mitochondrial network occurs. The viral antigens were located near the cell nucleus and they co-localized with mitochondria. In the later stage of HHV-1 and HHV-2 infection, presence of progeny virions within the cytoplasm and their co-localization with the mitochondrial structures were observed.

The accumulation of mitochondria in a close proximity of HHV-1 and HHV-2 antigens suggests that mitochondria are active and probably supply ATP for morphogenesis of progeny virions. Here, we found that the level of ATP in HHV-1- and HHV-2-infected neurons decreased, but it was not statistically significant, when compared to non-infected cells. Probably, despite alteration in mitochondrial physiology, these organelles were still able to produce ATP (Gregorczyk et al. 2018), therefore delaying induction of neuronal death.

We also observed that HHV-infection significantly affects the numbers of mitochondrial individuals and networks. Probably, longer rods were breaking up into smaller rods or punctuates and longer networks were breaking up into many smaller networks. As we suggested before (Gregorczyk et al. 2018), in neurons, mitochondrial division is important to transport mitochondria to sites where a high amount of energy is required.

Mitochondrial dynamics is a complex process, which involves the fission and fusion of mitochondrial outer and inner membranes. These processes organize the mitochondrial size and morphology, as well as their localization throughout the cells (Cid-Castro et al. 2018). Mitochondrial morphology is dependent on a proper balance between fusion and fission processes, which are coordinated by a systematized set of dynamin-related GTPases. In present work, we hypothesize that HHV-induced fragmentation is caused by enhanced fission. Although the present study shows that the level of Drp1 protein was unchanged during HHV-infection, the localization of this protein was altered. At the molecular level, we found that HHV-infection resulted in Drp1 activation, as indicated by a significantly increased association with mitochondria. During the late stage of infection with both HHV-1 and HHV-2, we found a progressive disintegration of the mitochondrial network, linked with accumulation of Drp1 protein and its co-localization with mitochondria. Drp1 is the master protein for mitochondrial fission, and it is initially positioned at the outer mitochondrial membranes by adaptor proteins. Drp1 leads membrane scission by forming a ring around the organelle to constrain the membranes resulting in mitochondrial shortening (Cid-Castro et al. 2018).

Deregulation of the mitochondrial fusion or fission has been also associated with defects in neuronal development and neuronal plasticity, both in ex vivo and in vivo models (Bertholet et al. 2016). In Drp1 mutant cultured neurons, abnormal mitochondrial distribution results in a compromised synapse formation (Ishihara et al. 2009). Drp1 defects have also been observed in Alzheimer’s disease patients (Kandimalla and Reddy 2016). The expression of Drp1 and its interaction with mitochondrial adaptors were markedly increased in SH5YSy cells treated by Aβ (Kuruva et al. 2017). In contrast, the inhibition of Drp1 interaction with its adaptors reduces the recruitment of Drp1 and prevents the mitochondrial fission and functional dysfunction induced by Aβ-42 (Joshi et al. 2017).

The evaluation of mitochondrial physiology indicates also that both HHV-1 and HHV-2 contributes to reduction of ΔΨ in neurons, especially during early stage of infection. Our results of mitochondrial fission in infected neurons confirmed that the loss of mitochondrial membrane potential is related with mitochondrial dysfunction and fragmentation. In addition, the reduction of ΔΨ is often a manifestation of apoptosis activation. Our results indicate that HHV-infected neurons exhibited a vitality decrease of about 20%.

Experimental evidence shows that the redox signaling is important for the mitochondrial dynamics in several cell types and that the levels of ROS are closely linked to the functioning of proteins involved in fission or fusion (Mailloux et al. 2013). The loss in the fusion and fission balance has been related to oxidative stress in neurons (Cid-Castro et al. 2018). It is also known that the loss of mitochondrial dynamics leads to an increase of ROS generation and a decrease in the ATP production (Guo et al. 2015). In general, high levels of ROS trigger mitochondrial fragmentation. This condition also leads to a modification of Drp1 activity. In an Alzheimer’s disease model, increased mitochondrial ROS levels lead to a shortening of mitochondria and an increase in Drp1 activation by the phosphorylation process (Cho et al. 2012). Moreover, Zorov et al. (2014) have shown that higher levels of ROS result in the loss of ΔΨ, which consequently leads to mitochondrial permeability transition and damage.

In the present work, we also examined the interaction between oxidative stress and HHV-1/HHV-2 infection. Our results are in agreement with data obtained by Valyi-Nagy and Dermody (2005) indicating that the direct effects of HHV-1 on neurons and host inflammatory response to infection can lead to oxidative damage due to increased formation of ROS. The role of ROS in the regulation of mitochondrial dynamics is critical for several neurodegenerative disorders. One of the earliest signals in the pathophysiological process of neurodegeneration is an imbalance of ROS. Based on our results, it can be suggested that the increased of ROS generated during HHV-infection triggers mitochondrial damage, manifested by the loss of ΔΨ and mitochondrial fragmentation. In turn, defective mitochondria generate more ROS which contributes to greater damage to mitochondria.

## Conclusions

Despite the evidence of morphological, biochemical, and molecular abnormalities in mitochondria in various tissues of patients with neurodegenerative disorders, the question “is a mitochondrial dysfunction a necessary step in HHV- induced neurodegeneration?” is still not fully clarified. Our research shows that from the functional perspective, HHV-1 and HHV-2 infection affected mitochondria at multiple levels, including a decrease of mitochondrial membrane potential and an increase of ROS level. HHV-infection caused an upregulation of mitochondrial fission that resulted in the mitochondrial fragmentation and abnormal distribution, which further contributed to mitochondrial and neuronal dysfunction. We suppose that in HHV-1- and HHV-2-infected neurons, mitochondrial division is important to transport mitochondria to the sites where a high amount of energy is required for the synthesis of progeny viruses.
